# Supplementary material for: Characterising the interventions designed to affect the reporting of musculoskeletal imaging: a scoping review protocol using the COM-B model
Source: BMJ Open. 2023 Nov 27;13(11):e072150. doi: 10.1136/bmjopen-2023-072150 (PMC10685969; doi:10.1136/bmjopen-2023-072150)
Supplement: Supplementary data [file bmjopen-2023-072150supp001.pdf]

Appendices

Appendix I: Search strategy

Ovid MEDLINE(R) ALL <1946 to December 14, 2022>

1 exp Musculoskeletal Pain/ or musculoskeletal.mp.

2 exp Patellofemoral Pain Syndrome/ or exp Back Pain/ or exp Low Back Pain/ or exp Shoulder Pain/ or exp Neck Pain/

3 osteoarthriti*.mp. or exp Osteoarthritis, Hip/ or exp Osteoarthritis/ or exp Osteoarthritis, Spine/ or exp Osteoarthritis, Knee/

4 spondylosis.mp. or exp Spondylosis/

5 (backache or lumbago or radicular or sciatica).mp.

6 ("leg pain" or "foot pain" or "heel pain" or "ankle pain" or "knee pain" or "patellofemoral pain" or "hip pain" or "arm pain" or "shoulder pain" or "subacromial pain" or "elbow pain" or "hand pain" or "wrist pain").mp.

7 exp chronic pain/ or "chronic pain".mp. or "persistent pain".mp.

8 exp Humans/

9 exp Animals/

10 8 and 9

11 9 not 10

12 "diagnostic imaging".mp. or exp Diagnostic Imaging/

13 exp Radiography/ or radiograph*.mp.

14 IMAGING.mp. or exp Magnetic Resonance Imaging/ or "magnetic resonance".mp. or MRI.mp.

15 exp Radiology/ or exp Radiologists/ or RADIOLOG*.mp. or exp Radiology Information Systems/

16 Tomography, X-Ray Computed/ or "COMPUTED TOMOGRAPHY".mp.

17 CT.mp.

18 (ultrasound or ultrasonograph*).mp.

19 exp X-Rays/

20 X?RAY.mp.

21 exp Education, Medical/ or exp Interprofessional Education/ or exp Patient Education Handout/ or exp Patient Education as Topic/ or exp Health Education/

22 exp Health Communication/

23 "HEALTH COMMUNICATION".mp.

24 "REPORTING STRATEG*".mp.

25 (EPIDEMIOLOGIC* adj2 (DATA or INFORMATION)).mp.

26 (PREVALENCE adj2 INFORMATION).mp

27 (PAIN adj2 EDUCATION).mp.

28 ("pain neurobiology" or "pain neurophysiology").mp.

29 Patient Education as Topic/ or PSYCHOEDUCATION*.mp.

30 (COMMUNICAT* adj5 ("IMAGing FINDINGs" or "IMAGing RESULTs" or "imaging report" or " MRI findings" or "MRI results" or "MRI REPORT*" or "X-RAY findings" or "X-Ray results" or "X-Ray REPORT" or "CT findings" or "CT REPORT" or "CT results" or "RADIOLOG* FINDINGS*" or "radiolog* results" or "radiolog* report")).

31 ((WRITTEN or PRINTED or ORAL) adj INFORMATION).mp.

32 (interpretation adj3 ("IMAGing FINDINGs" or "IMAGing RESULTs" or "imaging report" or " MRI findings" or "MRI results" or "MRI REPORT*" or "X-RAY findings" or "X-Ray results" or "X-Ray REPORT" or "CT findings" or "CT REPORT" or "CT results" or "RADIOLOG* FINDINGS*" or "radiolog* results" or "radiolog* report")).mp.

33 (REASSUR* adj3 ("IMAGing FINDINGs" or "IMAGing RESULTs" or "imaging report" or " MRI findings" or "MRI results" or "MRI REPORT*" or "X-RAY findings" or "X-Ray results" or "X-Ray REPORT" or "CT findings" or "CT REPORT" or "CT results" or "RADIOLOG* FINDINGS*" or "radiolog* results" or "radiolog* report")).mp.

34 (CONTEXTUALI* adj3 ("IMAGing FINDINGs" or "IMAGing RESULTs" or "imaging report" or " MRI findings" or "MRI results" or "MRI REPORT*" or "X-RAY findings" or "X-Ray results" or "X-Ray REPORT" or "CT findings" or "CT REPORT" or "CT results" or "RADIOLOG* FINDINGS*" or "radiolog* results" or "radiolog* report")).mp

35 ((perception or perceive) adj3 ("IMAGing FINDINGs" or "IMAGing RESULTs" or "imaging report" or " MRI findings" or "MRI results" or "MRI REPORT*" or "X-RAY findings" or "X-Ray

results" or "X-Ray REPORT" or "CT findings" or "CT REPORT" or "CT results" or "RADIOLOG* FINDINGS*" or "radiolog* results" or "radiolog* report")).mp.

36 (DIAGNOS* adj2 INFORMATION).mp.

37 (INTERVENTION* adj5 ("IMAGing FINDINGs" or "IMAGing RESULTs" or "imaging report" or " MRI findings" or "MRI results" or "MRI REPORT*" or "X-RAY findings" or "X-Ray results" or "X-Ray REPORT" or "CT findings" or "CT REPORT" or "CT results" or "RADIOLOG* FINDINGS*" or "radiolog* results" or "radiolog* report")).mp

38 BIOPSYCHOSOCIAL.mp.

39 (behavio?r adj2 intervention).mp.

40 "behavio?r change".mp.

41 exp cognitive behavioral therapy/ or "cognitive behavio?ral therapy".mp.

42 "cognitive functional therapy".mp.

43 exp Arthritis, Rheumatoid/

44 exp Neoplasms/ or sarcoma*.mp. or osteosarcoma*.mp. or tumo?r*.mp.

45 exp Musculoskeletal Diseases/cn, su [Congenital, Surgery]

46 exp Central Nervous System/

47 exp Central Nervous System Diseases/

48 exp Dentistry/

49 exp Tooth Diseases/

50 1 or 2 or 3 or 4 or 5 or 6 or 7

51 12 or 13 or 14 or 15 or 16 or 17 or 18 or 19 or 20

52 21 or 22 or 23 or 24 or 25 or 26 or 27 or 28 or 29 or 30 or 31 or 32 or 33 or 34 or 35 or 36 or 37 or 38 or 39 or 40 or 41 or 42

53 43 or 44 or 45 or 46 or 47 or 48 or 49

54 50 and 51 and 52

55 54 not 53

56 55 not 11

Appendix II: Data Extraction - Coding Rules

1. <u>Patient as mechanism of action</u>
   If the patient is given something that relates to the behaviour in question, e.g. a letter aiming to promote the robustness of the spine and benefits of movement, that they need to take with them to their appointment to discuss with their HCP code as: 7.1 - “Prompts/cues” Or 12.1 - “restructuring social environment” dependent on the format and extent of the information.

2. <u>Patient education materials</u>
   If patient education materials are delivered to an HCP to use with patients, then code as

   12.5 “adding objects to environment” and add additional code explaining nature of information eg. 5.1 information about health consequences, 7.1 “prompt/cue (poster)”, 9.2 “pro and cons”

   Consider additional coding if information comes with support eg. 3.2 - “social support”

   If the research team indicates that the patient education material eg. Booklet was intended to act as a reminder then also code as 7.1 - “Prompt cue”

   If Patient education material delivered directly to patients from researchers (or someone other than the clinician) – do not code.

3. <u>HCP Training</u>
   Code as 4.1 – “instruction on how to perform a behaviour”

   If involves practice then additionally code as 8.1 – “behavioural practice/rehearsal”

   Only code as 6.1 demonstration of the behaviour and 8.1 behavioural practice/rehearsal if the intervention explicitly states demonstration.

4. <u>Information provided to clinicians (relates closely to education and persuasion)</u>
   Prospective information e.g. “The consequence of harmful reporting is increasing fear and downstream costs” Code as 5.1 – “information about health consequences”.

   If information about emotional consequences code as 5.6 “ information about emotional cosequences”

   For retrospective information code as: 2.7 – “feedback on outcomes of behaviour”

5. <u>Amending/Withholding imaging findings (relates closely to restructuring and enablement)</u>
   12.1 Restructuring the physical environment

   May also involve 12.3 avoidance/reducing cues for the behaviour

   12.5 if adding objects to the environment

6. <u>Inclusion of case studies/patient vignettes</u>
   Code as 4.1 - “instruction on how to perform a behaviour unless there was explicit evidence of OR 6.1 “Demonstration of the behaviour” or 8.1 – “Behavioural Practice/Rehearsal”

Appendix III: Categorisation the use of theory (Painter et al, 2008)

1 - Informed by theory:

Theoretical framework or construct identified but no or limited/partial application of theoretical framework in study components and measures. For example, one study stated that the Health Belief Model (HBM) was used to develop intervention materials, but no applications of HBM constructs were explicitly described, and no HBM constructs were measured.

2 - Applied theory:

Theoretical framework specified and between one and half of the constructs applied in components of the study. An example was a study that described intervention materials as based on the HBM and addressed cues to action and perceived threat.

3 - Testing theory:

Theoretical framework specified; more than half of the theoretical constructs in intervention or descriptive/explanatory research were measured and explicitly tested, or two or more theories were explicitly compared to one another in the study. For example, a study stated that the HBM was used to develop intervention materials. The study used and measured five key constructs in the intervention and evaluation: perceived benefits and barriers, perceived susceptibility and severity, and cues to action. Mediation analyses were conducted with indicators of these constructs.

4 - Building or creating theory:

Developing new or revised/expanded theory using constructs specified, measured, and analysed in a study. For example, a study stated that a new hybrid theory was created to develop intervention materials. The new theory integrated HBM constructs, such as perceived benefits and barriers with other constructs such as group involvement and social norms. The study tested the intervention effects and the hypothesized theoretical constructs' mediating effects and examined each construct's contribution to the new extended theoretical framework.

It is worth noting that our determination of theory use along the continuum [will rely] on analysis of the article as published rather than the article's stated intent, title, or abstract. For example, if the authors of a given article claimed that they were applying a theory but only measured one construct, it would have been included in the "informed" by theory category. Stages of change are one core construct of the Transtheoretical Model (TTM), so if an article only used the stages of change construct from TTM, then it was "informed" by TTM [31].

Appendix IV. Example template for illustrating extracted BCT's

Number of BCT's			
Incompatible beliefs			
Framing/Reframing			
Adding objects to the environment			
Avoidance/reducing exposure to cues for the behaviour			
Credible source			
Behavioural practice/Rehearsal			
Prompts/cues			
Demonstration of the behaviour			
Information about health consequences			
Instruction on how to perform behaviour			
Action Planning			
Target: Patient/clinician			
Author			

Appendix V. Example template for illustrating relationship between BCT's and COM-B components

Motivation	Automatic	Emotions			
	Reflective	goals			
		optimism			
		Social professional role and identity			
		Beliefs about consequences			
		Beliefs about capabilities			
Opportunity	Physical	Environmental content and resources			
	Social	Social influences			
Capability	Psychological	Behavioural regulation			
		Memory attention and decision processes			
		Knowledge			
	Physical	Skills			
Author					
BCT					
