# Supplementary material for: Characterising the interventions designed to affect the reporting of musculoskeletal imaging: a scoping review protocol using the COM-B model
Source: BMJ Open. 2023 Nov 27;13(11):e072150. doi: 10.1136/bmjopen-2023-072150 (PMC10685969; doi:10.1136/bmjopen-2023-072150)
Supplement: Author's manuscript [file bmjopen-2023-072150.draft_revisions.pdf]

BMJ Open is committed to open peer review. As part of this commitment we make the peer review history of every article we publish publicly available.

When an article is published we post the peer reviewers' comments and the authors' responses online. We also post the versions of the paper that were used during peer review. These are the versions that the peer review comments apply to.

The versions of the paper that follow are the versions that were submitted during the peer review process. They are not the versions of record or the final published versions. They should not be cited or distributed as the published version of this manuscript.

BMJ Open is an open access journal and the full, final, typeset and author-corrected version of record of the manuscript is available on our site with no access controls, subscription charges or pay-per-view fees (http://bmjopen.bmj.com).

If you have any questions on BMJ Open's open peer review process please email info.bmjopen@bmj.com

BMJ Open

Characterising the interventions designed to affect the reporting of musculoskeletal imaging. A scoping review protocol using the Behaviour Change Wheel.

Journal:	*BMJ Open*
Manuscript ID	bmjopen-2023-072150
Article Type:	Protocol
Date Submitted by the Author:	02-Feb-2023
Complete List of Authors:	Kirby, Edward; University of Essex, Physiotherapy; Essex Partnership University NHS Foundation Trust<br>MacMillan, Andrew; University College of Osteopathy<br>X.W. Liew, Bernard ; University of Essex, School of Sport, Rehabilitation and Exercise Sciences; University of Essex<br>Brinkley, Andrew; University of Essex, School of Sport, Rehabilitation and Exercise Sciences<br>Bateman, Andrew; University of Essex, School of Health and Social Care
Keywords:	Back pain < ORTHOPAEDIC & TRAUMA SURGERY, MEDICAL EDUCATION & TRAINING, Chronic Pain, RADIOLOGY & IMAGING

SCHOLARONE™
Manuscripts

Characterising the interventions designed to affect the reporting of musculoskeletal imaging. A scoping review protocol using the Behaviour Change Wheel.

For peer review only

Authors

Edward Kirby[1] Andrew MacMillan[2] Bernard Liew[3] Andrew Brinkley [3] Andrew Bateman [3]

1. Essex Partnership University NHS Foundation Trust, 2. University College of Osteopathy, 3. University of Essex
2. Corresponding Author: Edward Kirby, edwardkirby@nhs.net

Abstract

Introduction:

Attributing musculoskeletal (MSK) pain to normal and commonly occurring imaging findings such as tendon, cartilage and spinal disc degeneration, has been shown to increase people's fear of movement, reduce their optimism about recovery and increase healthcare costs. Interventions seeking to reduce the negative effects of MSK imaging reporting have had little effect. To understand the ineffectiveness of these interventions, this study seeks to scope their behavioural targets, intended mechanisms of action and theoretical underpinnings. This information alongside known barriers to helpful reporting can enable researchers to refine or create new more targeted interventions .

Methods and Analysis:

The scoping review will be conducted in accordance with the JBI methodology for scoping reviews[25] and the Preferred Reporting Items for Systematic reviews and Meta-Analyses extension for

For peer review only - http://bmjopen.bmj.com/site/about/guidelines.xhtml

Scoping Reviews (PRISMA-ScR). Search terms will be devised by the research team. Searches of MEDLINE, EMBASE, CINAHL, AMED and PsycINFO from inception to current day will be performed. The review will include studies, which have developed or evaluated interventions targeting the reporting of musculoskeletal imaging. Studies targeting the diagnosis of serious causes of musculoskeletal pain will be excluded. Two independent authors will extract study participant data using predefined extraction templates and intervention details using the Template for Intervention Description and Replication (TIDieR) checklist. Interventions will be coded and mapped to the technique, mechanism of action and behavioural target according to the Capability, Opportunity, Motivation - Behaviour (COM-B) model categories. Any explicit models or theories used to inform the selection of interventions will be extracted and coded. The study characteristics, BCT's identified, behavioural targets according to the COM-B and context specific theories within the studies will be presented in narrative and table form.

Ethics and dissemination:

The information from this review will be used to inform an intervention design process seeking to improve the communication of imaging results. The results will also be disseminated through a peer-reviewed publication, conference presentations and stakeholder events.

Strengths and limitations of this Protocol:

- Novel use of a validated behaviour change framework to retrospectively highlight the targets of existing interventions.
- Comprehensive search strategy using a range of databases, hand searching and rigorous article screening performed by two independent authors and a third author to reconcile discrepancies.
- The quality of the study intervention reporting will determine the accuracy of the coding.

Introduction

Rationale

Data from the Global Burden of Disease Study indicate that Musculoskeletal (MSK) disorders (low back pain, neck pain, osteoarthritis, rheumatoid arthritis, and other musculoskeletal disorders) are the leading cause of disability globally in 2019 [1]. MSK conditions such as arthritis and back pain affected an estimated 18.8 million people across the UK in 2017 and accounted for more than 22% of the total burden of ill health in the UK with estimated cost to the NHS of over £5 billion annually [2]. Despite guidance on the management of the most common MSK conditions [3,4], studies have shown that in some cases Healthcare Practitioners (HCP's) may negatively affect their patients' disorder by influencing or perpetuating negative or unhelpful health beliefs [5].

For peer review only

Prior qualitative studies have identified that patients' primary goal for attending health services for MSK pain is a desire to identify a clear pathoanatomical cause [6] with the added belief that MSK imaging is a useful test to discover the source of the pain [7]. This may seem coherent based on the prevailing biomedical model, which determines that visible abnormalities on imaging are causally linked with pain [8]. Epidemiological studies however, have found that abnormalities, such as disc degeneration in the spine [9], degenerative cartilage tears in the knee [10] and tendon tears in the shoulder [11] are equally common in asymptomatic populations. The tenuous association between many common MSK imaging findings and pain means that Health Care Professionals (HCP's) have difficulty confidently providing a specific diagnosis to patients in such cases.

The perception held by some HCP's that their patients desire a clear diagnosis, combined with difficulty understanding reports and communicating diagnostic uncertainty have been cited as reasons for HCP's continuing to attribute the cause of MSK pain to findings of doubtful significance on imaging [6]. A number of negative consequences from speculative diagnostic labelling such as 'wear and tear', 'damage' and 'degeneration' have been reported. These include: increased fear of movement (FoM) [5], perception of poorer prognosis [12], reduced patient confidence in conservative management [13], withdrawal from valued life activities [14], impaired general health outcomes [15], and increases in unnecessary or low value treatments such as injections and surgery [12, 16].

In an attempt to prevent harmful MSK image reporting behaviours, studies have evaluated the withholding of imaging reports [15], reassurance about the lack of serious pathology [17] and the insertion of information about the prevalence of findings in the pain free population [18]. A recent systematic review however, found little evidence of effectiveness for any of the interventions [19]. While these interventions attempted to change clinician-reporting behaviours, they did not outline a thorough intervention design process informed by theory. The National Institute for Health and Care Research (NIHR) and Medical Research Council (MRC) [20] recommend using behavioural science in the development of interventions to improve effectiveness and the lack of such a process in these studies may have contributed to their lack of effectiveness.

One approach to developing an effective behaviour change intervention for MSK reporting would be to utilise The Behaviour Change Wheel (BCW) [21]. The BCW was developed from 19 different behaviour change frameworks by synthesising their common features to create a unifying behavioural framework. It outlines a process that uses the Capability, Opportunity, Motivation - Behaviour (COM-B) Model to identify behavioural targets, which then link to nine intervention function options, which can be selected based on the COM-B analysis [21]. In the absence of an explicit design process, The BCW can also be used as a framework to retrospectively highlight the behavioural targets of prior interventions. For example, Hall et al (2021) [22], analysed existing interventions to ascertain which BCTs have been used to improve adherence to evidence-based low back pain imaging requests and found that the majority of included studies "lacked the use of any theory or framework to inform their intervention design". By using the BCW as a framework to map the interventions to known barriers to guideline adherence, they found that the interventions "failed to target known physician-reported barriers to following LBP imaging guidelines".

Using the BCW to map existing MSK imaging reporting interventions to their behavioural targets in this way, will elucidate the congruence of existing interventions with the known barriers and enablers of helpful reporting. This information can enable researchers to modify, adapt or develop new and potentially more targeted and effective reporting interventions.

A preliminary search of PROSPERO, Open Science Framework, MEDLINE, the Cochrane Database of Systematic Reviews and the JBI Evidence Synthesis indicates that no study has attempted to map the behaviour change techniques used or the theory underpinning interventions designed to affect the communication of MSK imaging findings.

Objectives

The objective of this review is to discover which Behaviour Change Techniques (BCT's) have been employed, what the behavioural targets were and which behavioural theories and theoretical models underpinned the interventions designed to affect MSK image reporting. This information may provide direction for the design of future interventions, by highlighting strategies that show promise and strategies that have not been explored.

Methods

The proposed scoping review will be conducted in accordance with the JBI methodology for scoping reviews [24] and the Preferred Reporting Items for Systematic reviews and Meta-Analyses extension for Scoping Reviews (PRISMA-ScR) [25]. The protocol was registered with the Open Science Framework https://doi.org/10.17605/OSF.IO/ECYS8. Any deviations from the protocol will be reported and justified in the methods section of the final review manuscript.

Eligibility Criteria

The review will include Studies that have developed or evaluated interventions to target the communication of musculoskeletal imaging findings. Studies in any healthcare setting worldwide and published in any language will be included, where languages other than English will be translated. Studies that include multiple interventions or broad treatment approaches will be included if it was possible to isolate specific BCTs intended to affect the communication of imaging findings within them. Studies using qualitative methods to develop interventions will be included if their findings identify a target for interventions. The review will exclude studies focussing on serious or specific known causes of musculoskeletal pain such as fracture, malignancy, infection and inflammatory arthritis.

This scoping review will consider both experimental and quasi-experimental study designs including randomized controlled trials, non-randomized controlled trials, before and after studies and interrupted time-series studies, cluster randomised trials, non-randomised cluster trials, controlled and uncontrolled before-after studies and cross sectional studies. Qualitative studies will be included for extraction of behavioural and context specific theories.

For peer review only - http://bmjopen.bmj.com/site/about/guidelines.xhtml

Information sources and Search strategy

The search strategy will aim to locate published studies. A three-step search strategy will be utilized in this review. First, an initial limited search of MEDLINE (PubMed) and CINAHL (EBSCO) was undertaken to identify articles on the topic. The text words contained in the titles and abstracts of relevant articles, and the index terms used to describe the articles were used to develop a full search strategy for MEDLINE, EMBASE, CINAHL, AMED and PsycINFO (see Appendix I). We will email experts in the field of imaging and LBP to identify any studies that may have been missed by the search. The search strategy, including all identified keywords and index terms, will be adapted for each included database and/or information source. The reference list of all included sources of evidence will be screened for additional studies. A specialist healthcare librarian will review the search strategy.

Study selection

Following the search, all identified citations will be collated and uploaded into Refworks [Proquest LLC, Michigan USA] and duplicates removed. Following a pilot test, titles and abstracts will then be screened by two or more independent reviewers for assessment against the inclusion criteria for the review. Potentially relevant sources will be retrieved in full and assessed in detail against the inclusion criteria by two or more independent reviewers. Reasons for exclusion of papers at full text that do not meet the inclusion criteria will be recorded and reported in the systematic review. Any disagreements that arise between the reviewers at each stage of the selection process will be resolved through discussion, or with an additional reviewer/s. The results of the search and the study inclusion process will be reported in full in the final systematic review and presented in a Preferred Reporting Items for Systematic Reviews and Meta-analyses (PRISMA) flow diagram [26]

Data extraction

Two reviewers will independently extract the study characteristics (year, country, setting, design, patient numbers (n), outcome measures used and MSK area) and intervention information using the Template for Intervention Description and Replication (TIDieR) [27].

The TIDieR template [27] will be used to characterise the intervention. A draft coding manual will be created based on the BCT taxonomy v1 [see Appendix II] and piloted. Coding will based on principles outlined in Lorencato et al (2013) [28]. The draft data extraction tool will be modified and revised as necessary during the process of extracting data from each included evidence source. Any coding disagreements that arise between the reviewers will be resolved through discussion, or with a third reviewer. Authors of papers will be contacted to request missing or additional data, where required.

The Theory and Techniques Tool (TATT) (available at: https://theoryandtechniquetool. humanbehaviourchange.org/tool) will be used to map BCTs to their corresponding mechanisms of action in the TDF. The Theory and Techniques tool is a map of 74 BCTs linking to 26 mechanisms of action and attributing strength to the association based on research and expert consensus.

For peer review only

Question 2 on the template - "describe the use of any rationale, theory or goal of the elements essential to the intervention" will be used to extract both evidence of behavioural theory used and context specific models. The Painter criteria [31] will then be applied to the details extracted. This categorisation [see Appendix III] distinguishes the use of theory into the following categories: (i) informed by theory, (ii) applied theory, (iii) testing theory or (iv) building or creating theory.

Data presentation

The Study characteristics, BCT's identified, behavioural targets (according to the COM-B) and context specific theories within the studies will be presented in narrative and table form.

Ethics and dissemination

This review will be the first step to formally identify which BCT's have been employed, what the behavioural targets were and which behavioural theories and theoretical models underpinned the interventions designed to affect MSK image reporting. This information will be used to inform an intervention design process seeking to improve the communication of imaging results. The results will be disseminated through a peer-reviewed publication, conference presentations and stakeholder events.

Patient and Public Involvement (PPI)

Patients with experience of receiving MSK imaging reports were invited to participate in individual sessions initially by advertising in person and using study flyers within radiology departments and GP surgeries. These initial sessions sought to explore peoples' experience of imaging report communication and the ways that this could be improved, such as the setting, personnel involved and resources that would be helpful to them. These meetings highlighted clinical behaviours that were discordant with patient preference. Based on this information it was deemed necessary to investigate the barriers to providing helpful communication of reports and the current review of whether existing interventions had targeted these. Further PPI group sessions are planned to discuss the results of this review and to have input into the design and implementation of further work seeking to improve the communication of MSK imaging findings.

For peer review only

Author Contributions

EK designed and wrote the protocol with planning and design input from BL and ABr and ABa. Studies will be screened by EK and AM. Data will be extracted by EK and AM. Data will be coded by EK and AB. AM, BL, ABr and Aba will review and make amendments to drafts of the manuscript and approve the final draft.

Funding

EK is an ICA Pre-doctoral Clinical and Practitioner Academic Fellow supported by Health Education England and the National Institute for Health and Care Research."

Declarations

The views expressed in this publication are those of the author(s) and not necessarily those of the NHS, the National Institute for Health and Care Research or the Department of Health and Social Care

Conflicts of interest

There is no conflict of interest in this project

References

1. Wu A, March L, Zheng X, Huang J, Wang X, Zhao J, et al. Global low back pain prevalence and years lived with disability from 1990 to 2017: estimates from the Global Burden of Disease Study 2017. Annals of translational medicine. 2020;8(6).

2. Global Burden of Disease Collaborative Network. Global Burden of Disease Study 2017 (GBD 2017) Results. Seattle: Institute for Health Metrics and Evaluation (IHME); 2018.

3. National Institute for Health and Care Excellence. Osteoarthritis in over 16s: diagnosis and management [Internet]. UK: National Institute for Health and Care Excellence; 2022 [cited 2022 Nov 17]. Available from: https://www.nice.org.uk/guidance/ng226

4. National Institute for Health and Care Excellence 2016. Low back pain and sciatica in over 16s: assessment and management [Internet]. UK: National Institute for Health and Care Excellence.; 2016 [cited 2022 Nov 17]. Available from: https://www.nice.org.uk/guidance/ng59

For peer review only

5. Lin IB, O'Sullivan PB, Coffin JA, Mak DB, Toussaint S, Straker LM. Disabling chronic low back pain as an iatrogenic disorder: a qualitative study in Aboriginal Australians. BMJ open. 2013;3(4).

6. Slade SC, Molloy E, Keating JL. The dilemma of diagnostic uncertainty when treating people with chronic low back pain: a qualitative study. Clinical rehabilitation. 2012;26(6):69.

7. Sharma S, Traeger AC, Reed B, Hamilton M, O'Connor DA, Hoffmann TC, et al. Clinician and patient beliefs about diagnostic imaging for low back pain: a systematic qualitative evidence synthesis. BMJ open. 2020;10(8).

8. Farre A, Rapley T. The New Old (and Old New) Medical Model: Four Decades Navigating the Biomedical and Psychosocial Understandings of Health and Illness. Healthcare (Basel, Switzerland). 2017;5(4).

9. Brinjikji W, Luetmer PH, Comstock B, Bresnahan BW, Chen LE, Deyo RA, et al. Systematic literature review of imaging features of spinal degeneration in asymptomatic populations. AJNR American journal of neuroradiology. 2015;36(4):6.

10. Horga LM, Hirschmann AC, Henckel J, Fotiadou A, Di Laura A, Torlasco C, et al. Prevalence of abnormal findings in 230 knees of asymptomatic adults using 3.0 T MRI. Skeletal radiology. 2020;49(7):1107.

11. Barreto RPG, Braman JP, Ludewig PM, Ribeiro LP, Camargo PR. Bilateral magnetic resonance imaging findings in individuals with unilateral shoulder pain. Journal of shoulder and elbow surgery. 2019;28(9):1706.

12. O'Keeffe M, Ferreira GE, Harris IA, Darlow B, Buchbinder R, Traeger AC, et al. Effect of diagnostic labelling on management intentions for non-specific low back pain: A randomized scenario-based experiment. European journal of pain (London, England). 2022;26(7):1545.

13. Malliaras P, Rathi S, Burstein F, Watt L, Ridgway J, King C, et al. "Physio's not going to repair a torn tendon": patient decision-making related to surgery for rotator cuff related shoulder pain. Disability and rehabilitation. 2022;44(14):3693.

14. Bunzli S, Watkins R, Smith A, Schutze R, O'Sullivan P. Lives on hold: a qualitative synthesis exploring the experience of chronic low-back pain. The Clinical journal of pain. 2013;29(10):16.

15. Ash LM, Modic MT, Obuchowski NA, Ross JS, Brant-Zawadzki MN, Grooff PN. Effects of diagnostic information, per se, on patient outcomes in acute radiculopathy and low back pain. AJNR American journal of neuroradiology. 2008;29(6):103.

16. Graves JM, Fulton-Kehoe D, Jarvik JG, Franklin GM. Health care utilization and costs associated with adherence to clinical practice guidelines for early magnetic resonance imaging among workers with acute occupational low back pain. Health services research. 2014;49(2):65.

For peer review only

17. Rajasekaran S, Dilip Chand Raja S, Pushpa BT, Ananda KB, Ajoy Prasad S, Rishi MK. The catastrophization effects of an MRI report on the patient and surgeon and the benefits of “clinical reporting”: results from an RCT and blinded trials. European spine journal : official publication of the European Spine Society, the European Spinal Deformity Society, and the European Section of the Cervical Spine Research Society. 2021;30(7):2081.

18. Jarvik JG, Meier EN, James KT, Gold LS, Tan KW, Kessler LG, et al. The Effect of Including Benchmark Prevalence Data of Common Imaging Findings in Spine Image Reports on Health Care Utilization Among Adults Undergoing Spine Imaging: A Stepped-Wedge Randomized Clinical Trial. JAMA network open. 2020;3(9).

19. Witherow JL, Jenkins HJ, Elliott JM, Ip GH, Maher CG, Magnussen JS, et al. Characteristics and Effectiveness of Interventions That Target the Reporting, Communication, or Clinical Interpretation of Lumbar Imaging Findings: A Systematic Review. AJNR American journal of neuroradiology. 2022;43(3):500.

20. Skivington K, Matthews L, Simpson SA, Craig P, Baird J, Blazeby JM, et al. A new framework for developing and evaluating complex interventions: update of Medical Research Council guidance. BMJ (Clinical research ed). 2021;374.

21. Michie S AL. The Behaviour Change Wheel - A Guide to Designing Interventions. 1st ed. Great Britain: Silverback Publishing; 2014. 329 p.

For peer review only

22. Hall A, Richmond H, Pike A, Lawrence R, Etchegary H, Swab M, et al. What behaviour change techniques have been used to improve adherence to evidence-based low back pain imaging?. Implementation science : IS. 2021;16(1).

23. Michie S, Hyder N, Walia A, West R. Development of a taxonomy of behaviour change techniques used in individual behavioural support for smoking cessation. Addictive behaviors. 2011;36(4):9.

24. Aromataris E MZ. J. JBI Manual for Evidence Synthesis. 1st ed. Australia: JBI; 2020.

25. Tricco AC, Lillie E, Zarin W, O'Brien KK, Colquhoun H, Levac D, et al. PRISMA Extension for Scoping Reviews (PRISMA-ScR): Checklist and Explanation. Annals of internal medicine. 2018;169(7):473.

26. Moher D, Liberati A, Tetzlaff J, Altman DG, PRISMA Group. Preferred reporting items for systematic reviews and meta-analyses: the PRISMA statement. BMJ (Clinical research ed). 2009;339.

27. Hoffmann TC, Glasziou PP, Boutron I, Milne R, Perera R, Moher D, et al. Better reporting of interventions: template for intervention description and replication (TIDieR) checklist and guide. BMJ (Clinical research ed). 2014;348.

28. Lorencatto F, West R, Seymour N, Michie S. Developing a method for specifying the components of behavior change interventions in practice: the example of smoking cessation. Journal of consulting and clinical psychology. 2013;81(3):44.

29. Painter JE, Borba CPC, Hynes M, Mays D, Glanz K. The use of theory in health behavior research from 2000 to 2005: A systematic review. Annals of Behavioral Medicine [Internet]. 2008 Jun;35(3):362. Available from: https://www.proquest.com/scholarly-journals/use-theory-health-behavior-research-2000-2005/docview/837460954/se-2?accountid=47999

For peer review only

Appendices

Appendix I: Search strategy

Ovid MEDLINE(R) ALL <1946 to December 14, 2022>

1	exp Musculoskeletal Pain/ or musculoskeletal.mp.
2	exp Patellofemoral Pain Syndrome/ or exp Back Pain/ or exp Low Back Pain/ or exp Shoulder Pain/ or exp Neck Pain/
3	osteoarthriti*.mp. or exp Osteoarthritis, Hip/ or exp Osteoarthritis/ or exp Osteoarthritis, Spine/ or exp Osteoarthritis, Knee/
4	spondylosis.mp. or exp Spondylosis/
5	(backache or lumbago or radicular or sciatica).mp.
6	("leg pain" or "foot pain" or "heel pain" or "ankle pain" or "knee pain" or "patellofemoral pain" or "hip pain" or "arm pain" or "shoulder pain" or "subacromial pain" or "elbow pain" or "hand pain" or "wrist pain").mp.
7	exp chronic pain/ or "chronic pain".mp. or "persistent pain".mp.
8	exp Humans/
9	exp Animals/
10	8 and 9
11	9 not 10
12	"diagnostic imaging".mp. or exp Diagnostic Imaging/
13	exp Radiography/ or radiograph*.mp.
14	IMAGING.mp. or exp Magnetic Resonance Imaging/ or "magnetic resonance".mp. or MRI.mp.
15	exp Radiology/ or exp Radiologists/ or RADIOLOG*.mp. or exp Radiology Information Systems/
16	Tomography, X-Ray Computed/ or "COMPUTED TOMOGRAPHY".mp.
17	CT.mp.
18	(ultrasound or ultrasonograph*).mp.

For peer review only

19	exp X-Rays/
20	X?RAY.mp.
21	exp Education, Medical/ or exp Interprofessional Education/ or exp Patient Education Handout/ or exp Patient Education as Topic/ or exp Health Education/
22	exp Health Communication/
23	"HEALTH COMMUNICATION".mp.
24	"REPORTING STRATEG*".mp.
25	(EPIDEMIOLOGIC* adj2 (DATA or INFORMATION)).mp.
26	(PREVALENCE adj2 INFORMATION).mp
27	(PAIN adj2 EDUCATION).mp.
28	("pain neurobiology" or "pain neurophysiology").mp.
29	Patient Education as Topic/ or PSYCHOEDUCATION*.mp.
30	(COMMUNICAT* adj5 ("IMAGing FINDINGs" or "IMAGing RESULTs" or "imaging report" or " MRI findings" or "MRI results" or "MRI REPORT*" or "X-RAY findings" or "X-Ray results" or "X-Ray REPORT" or "CT findings" or "CT REPORT" or "CT results" or "RADIOLOG* FINDINGS*" or "radiolog* results" or "radiolog* report")).
31	((WRITTEN or PRINTED or ORAL) adj INFORMATION).mp.
32	(interpretation adj3 ("IMAGing FINDINGs" or "IMAGing RESULTs" or "imaging report" or " MRI findings" or "MRI results" or "MRI REPORT*" or "X-RAY findings" or "X-Ray results" or "X-Ray REPORT" or "CT findings" or "CT REPORT" or "CT results" or "RADIOLOG* FINDINGS*" or "radiolog* results" or "radiolog* report")).mp.
33	(REASSUR* adj3 ("IMAGing FINDINGs" or "IMAGing RESULTs" or "imaging report" or " MRI findings" or "MRI results" or "MRI REPORT*" or "X-RAY findings" or "X-Ray results" or "X-Ray REPORT" or "CT findings" or "CT REPORT" or "CT results" or "RADIOLOG* FINDINGS*" or "radiolog* results" or "radiolog* report")).mp.
34	(CONTEXTUALI* adj3 ("IMAGing FINDINGs" or "IMAGing RESULTs" or "imaging report" or " MRI findings" or "MRI results" or "MRI REPORT*" or "X-RAY findings" or "X-Ray results" or "X-Ray REPORT" or "CT findings" or "CT REPORT" or "CT results" or "RADIOLOG* FINDINGS*" or "radiolog* results" or "radiolog* report")).mp
35	((perception or perceive) adj3 ("IMAGing FINDINGs" or "IMAGing RESULTs" or "imaging report" or " MRI findings" or "MRI results" or "MRI REPORT*" or "X-RAY findings" or "X-Ray

For peer review only

	results" or "X-Ray REPORT" or "CT findings" or "CT REPORT" or "CT results" or "RADIOLOG* FINDINGS*" or "radiolog* results" or "radiolog* report")).mp.
36	(DIAGNOS* adj2 INFORMATION).mp.
37	(INTERVENTION* adj5 ("IMAGing FINDINGs" or "IMAGing RESULTs" or "imaging report" or " MRI findings" or "MRI results" or "MRI REPORT*" or "X-RAY findings" or "X-Ray results" or "X-Ray REPORT" or "CT findings" or "CT REPORT" or "CT results" or "RADIOLOG* FINDINGS*" or "radiolog* results" or "radiolog* report")).mp
38	BIOPSYCHOSOCIAL.mp.
39	(behavio?r adj2 intervention).mp.
40	"behavio?r change".mp.
41	exp cognitive behavioral therapy/ or "cognitive behavio?ral therapy".mp.
42	"cognitive functional therapy".mp.
43	exp Arthritis, Rheumatoid/
44	exp Neoplasms/ or sarcoma*.mp. or osteosarcoma*.mp. or tumo?r*.mp.
45	exp Musculoskeletal Diseases/cn, su [Congenital, Surgery]
46	exp Central Nervous System/
47	exp Central Nervous System Diseases/
48	exp Dentistry/
49	exp Tooth Diseases/
50	1 or 2 or 3 or 4 or 5 or 6 or 7
51	12 or 13 or 14 or 15 or 16 or 17 or 18 or 19 or 20
52	21 or 22 or 23 or 24 or 25 or 26 or 27 or 28 or 29 or 30 or 31 or 32 or 33 or 34 or 35 or 36 or 37 or 38 or 39 or 40 or 41 or 42
53	43 or 44 or 45 or 46 or 47 or 48 or 49
54	50 and 51 and 52
55	54 not 53
56	55 not 11

For peer review only

Appendix II: Data Extraction - Coding Rules

1. Patient as mechanism of action
   If the patient is given something that relates to the behaviour in question, e.g. a letter aiming to promote the robustness of the spine and benefits of movement, that they need to take with them to their appointment to discuss with their HCP code as: 7.1 - "Prompts/cues" Or 12.1 - "restructuring social environment" dependent on the format and extent of the information.

2. Patient education materials
   If patient education materials are delivered to an HCP to use with patients, then code as

   12.5 "adding objects to environment" and add additional code explaining nature of information eg. 5.1 information about health consequences, 7.1 "prompt/cue (poster)", 9.2 "pro and cons"

   Consider additional coding if information comes with support eg. 3.2 - "social support"

   If the research team indicates that the patient education material eg. Booklet was intended to act as a reminder then also code as 7.1 - "Prompt cue"

   If Patient education material delivered directly to patients from researchers (or someone other than the clinician) – do not code.

3. HCP Training
   Code as 4.1 – "instruction on how to perform a behaviour"

   If involves practice then additionally code as 8.1 – "behavioural practice/rehearsal"

   Only code as 6.1 demonstration of the behaviour and 8.1 behavioural practice/rehearsal if the intervention explicitly states demonstration.

4. Information provided to clinicians (relates closely to education and persuasion)
   Prospective information e.g. "The consequence of harmful reporting is increasing fear and downstream costs" Code as 5.1 – "information about health consequences".

   If information about emotional consequences code as 5.6 " information about emotional cosequences"

   For retrospective information code as: 2.7 – "feedback on outcomes of behaviour"

5. Amending/Withholding imaging findings (relates closely to restructuring and enablement)
   12.1 Restructuring the physical environment

   May also involve 12.3 avoidance/reducing cues for the behaviour

   12.5 if adding objects to the environment

6. Inclusion of case studies/patient vignettes
   Code as 4.1 - "instruction on how to perform a behaviour unless there was explicit evidence of OR 6.1 "Demonstration of the behaviour" or 8.1 – "Behavioural Practice/Rehearsal"

For peer review only

Appendix III: Categorisation the use of theory (Painter et al, 2008)

1 - Informed by theory:

Theoretical framework or construct identified but no or limited/partial application of theoretical framework in study components and measures. For example, one study stated that the Health Belief Model (HBM) was used to develop intervention materials, but no applications of HBM constructs were explicitly described, and no HBM constructs were measured.

2 - Applied theory:

Theoretical framework specified and between one and half of the constructs applied in components of the study. An example was a study that described intervention materials as based on the HBM and addressed cues to action and perceived threat.

3 - Testing theory:

Theoretical framework specified; more than half of the theoretical constructs in intervention or descriptive/explanatory research were measured and explicitly tested, or two or more theories were explicitly compared to one another in the study. For example, a study stated that the HBM was used to develop intervention materials. The study used and measured five key constructs in the intervention and evaluation: perceived benefits and barriers, perceived susceptibility and severity, and cues to action. Mediation analyses were conducted with indicators of these constructs.

4 - Building or creating theory:

Developing new or revised/expanded theory using constructs specified, measured, and analysed in a study. For example, a study stated that a new hybrid theory was created to develop intervention materials. The new theory integrated HBM constructs, such as perceived benefits and barriers with other constructs such as group involvement and social norms. The study tested the intervention effects and the hypothesized theoretical constructs' mediating effects and examined each construct's contribution to the new extended theoretical framework.

It is worth noting that our determination of theory use along the continuum [will rely] on analysis of the article as published rather than the article's stated intent, title, or abstract. For example, if the authors of a given article claimed that they were applying a theory but only measured one construct, it would have been included in the "informed" by theory category. Stages of change are one core construct of the Transtheoretical Model (TTM), so if an article only used the stages of change construct from TTM, then it was "informed" by TTM [31].

For peer review only

BMJ Open

Characterising the interventions designed to affect the reporting of musculoskeletal imaging. A scoping review protocol using the Behaviour Change Wheel.

Journal:	*BMJ Open*
Manuscript ID	bmjopen-2023-072150.R1
Article Type:	Protocol
Date Submitted by the Author:	29-Jun-2023
Complete List of Authors:	Kirby, Edward; University of Essex, Physiotherapy; Essex Partnership University NHS Foundation Trust<br>MacMillan, Andrew; University College of Osteopathy<br>X.W. Liew, Bernard ; University of Essex, School of Sport, Rehabilitation and Exercise Sciences; University of Essex<br>Brinkley, Andrew; University of Essex, School of Sport, Rehabilitation and Exercise Sciences<br>Bateman, Andrew; University of Essex, School of Health and Social Care
**Primary Subject Heading**:	Radiology and imaging
Secondary Subject Heading:	Communication, Medical education and training, Rehabilitation medicine
Keywords:	Back pain < ORTHOPAEDIC & TRAUMA SURGERY, MEDICAL EDUCATION & TRAINING, Chronic Pain, RADIOLOGY & IMAGING, ORTHOPAEDIC & TRAUMA SURGERY

SCHOLARONE™
Manuscripts

Characterising the interventions designed to affect the reporting of musculoskeletal imaging. A scoping review protocol using the Behaviour Change Wheel.

Authors

Edward Kirby[1] Andrew MacMillan[2] Bernard Liew[3] Andrew Brinkley [3] Andrew Bateman [3]

1. Essex Partnership University NHS Foundation Trust, 2. University College of Osteopathy, 3. University of Essex
2. Corresponding Author: Edward Kirby, edwardkirby@nhs.net

Abstract

Introduction:

Attributing musculoskeletal (MSK) pain to normal and commonly occurring imaging findings such as tendon, cartilage and spinal disc degeneration, has been shown to increase people's fear of movement, reduce their optimism about recovery and increase healthcare costs. Interventions seeking to reduce the negative effects of MSK imaging reporting have had little effect. To understand the ineffectiveness of these interventions, this study seeks to scope their behavioural targets, intended mechanisms of action and theoretical underpinnings. This information alongside known barriers to helpful reporting can enable researchers to refine or create new more targeted interventions .

Methods and Analysis:

The scoping review will be conducted in accordance with the JBI methodology for scoping reviews and the Preferred Reporting Items for Systematic reviews and Meta-Analyses extension for Scoping Reviews (PRISMA-ScR). Search terms will be devised by the research team. Searches of MEDLINE, EMBASE, CINAHL, AMED and PsycINFO from inception to current day will be performed. The review will include studies, which have developed or evaluated interventions targeting the reporting of musculoskeletal imaging. Studies targeting the diagnosis of serious causes of musculoskeletal pain will be excluded. Two independent authors will extract study participant data using predefined extraction templates and intervention details using the Template for Intervention

For peer review only

Description and Replication (TIDieR) checklist. Interventions will be coded and mapped to the technique, mechanism of action and behavioural target according to the Capability, Opportunity, Motivation - Behaviour (COM-B) model categories. Any explicit models or theories used to inform the selection of interventions will be extracted and coded. The study characteristics, Behaviour Change techniques (BCT's) identified, behavioural targets according to the COM-B and context specific theories within the studies will be presented in narrative and table form.

Ethics and dissemination:

The information from this review will be used to inform an intervention design process seeking to improve the communication of imaging results. The results will also be disseminated through a peer-reviewed publication, conference presentations and stakeholder events.

Strengths and limitations of this Protocol:

- Novel use of a validated behaviour change framework to retrospectively highlight the targets of existing interventions.
- Comprehensive search strategy using a range of databases, hand searching and rigorous article screening performed by two independent authors and a third author to reconcile discrepancies.
- The communication of imaging reporting may be an embedded component of broader treatment approaches and therefore difficult to identify during literature searching.
- The quality of the coding may be limited by the accuracy of the intervention reporting.

Introduction

Rationale

Data from the Global Burden of Disease Study indicate that Musculoskeletal (MSK) disorders (low back pain, neck pain, osteoarthritis, rheumatoid arthritis, and other musculoskeletal disorders) are the leading cause of disability globally in 2019. (1) MSK conditions such as arthritis and back pain affected an estimated 18.8 million people across the UK in 2017 and accounted for more than 22% of the total burden of ill health in the UK with estimated cost to the NHS of over £5 billion annually. (2) Despite guidance on the management of the most common MSK conditions, (3,4) studies have shown that in some cases Healthcare Practitioners (HCP's) may negatively affect their patients' disorder by influencing or perpetuating negative or unhelpful health beliefs. (5)

Prior qualitative studies have identified that patients' primary goal for attending health services for MSK pain is a desire to identify a clear pathoanatomical cause (6) with the added belief that MSK imaging is a useful test to discover the source of the pain. (7) This may seem coherent based on the

For peer review only - http://bmjopen.bmj.com/site/about/guidelines.xhtml

prevailing biomedical model, which determines that visible abnormalities on imaging are causally linked with pain. (8) Epidemiological studies however, have found that abnormalities, such as disc degeneration in the spine, (9) degenerative cartilage tears in the knee (10) and tendon tears in the shoulder (11) are equally common in asymptomatic populations. The tenuous association between many common MSK imaging findings and pain means that Health Care Professionals (HCP's) have difficulty confidently providing a specific diagnosis to patients in such cases.

The perception held by some HCP's that their patients desire a clear diagnosis, combined with difficulty understanding reports and communicating diagnostic uncertainty have been cited as reasons for HCP's continuing to attribute the cause of MSK pain to findings of doubtful significance on imaging. (6) A number of negative consequences from speculative diagnostic labelling such as 'wear and tear', 'damage' and 'degeneration' have been reported. These include: increased fear of movement (FoM), (5) perception of poorer prognosis, (12) reduced patient confidence in conservative management, (13) withdrawal from valued life activities, (14) impaired general health outcomes, (15) and increases in unnecessary or low value treatments such as injections and surgery. (12, 16)

In an attempt to prevent harmful MSK image reporting behaviours, studies have evaluated the withholding of imaging reports,(15) reassurance about the lack of serious pathology (17) and the insertion of information about the prevalence of findings in the pain free population. (18) A recent systematic review however, found little evidence of effectiveness for any of the interventions. (19) While these interventions attempted to change clinician-reporting behaviours, they did not outline a thorough intervention design process informed by theory. The National Institute for Health and Care Research (NIHR) and Medical Research Council (MRC) (20) recommend using behavioural science in the development of interventions to improve effectiveness and the lack of such a process in these studies may have contributed to their lack of effectiveness.

One approach to developing an effective behaviour change intervention for MSK reporting would be to utilise The Behaviour Change Wheel (BCW). (21) The BCW was developed from 19 different behaviour change frameworks by synthesising their common features to create a unifying behavioural framework. It outlines a process that uses the Capability, Opportunity, Motivation - Behaviour (COM-B) Model to identify behavioural targets, which then link to nine intervention function options, which can be selected based on the COM-B analysis. (21) In the absence of an explicit design process, The BCW can also be used as a framework to retrospectively highlight the behavioural targets of prior interventions. For example, Hall et al (2021),(22) analysed existing interventions to ascertain which BCTs (23) have been used to improve adherence to evidence-based low back pain imaging requests and found that the majority of included studies "lacked the use of any theory or framework to inform their intervention design". By using the BCW as a framework to map the interventions to known barriers to guideline adherence, they found that the interventions "failed to target known physician-reported barriers to following LBP imaging guidelines".

Using the BCW to map existing MSK imaging reporting interventions to their behavioural targets in this way, will elucidate the congruence of existing interventions with the known barriers and enablers of helpful reporting. This information can enable researchers to modify, adapt or develop new and potentially more targeted and effective reporting interventions.

For peer review only

A preliminary search of PROSPERO, Open Science Framework, MEDLINE, the Cochrane Database of Systematic Reviews and the JBI Evidence Synthesis indicates that no study has attempted to map the behaviour change techniques used or the theory underpinning interventions designed to affect the communication of MSK imaging findings.

Objectives

The objective of this review is to discover which BCT's have been employed, what the behavioural targets were and which behavioural theories and theoretical models underpinned the interventions designed to affect MSK image reporting. This information may provide direction for the design of future interventions, by highlighting strategies that show promise and strategies that have not been explored.

Methods

The proposed scoping review will be conducted in accordance with the JBI methodology for scoping reviews (24) and the Preferred Reporting Items for Systematic reviews and Meta-Analyses extension for Scoping Reviews (PRISMA-ScR). (25) The protocol was registered with the Open Science Framework https://doi.org/10.17605/OSF.IO/ECYS8. The planned start date for the study is 17th July and is planned to end on October 2023. Any deviations from the protocol will be reported and justified in the methods section of the final review manuscript.

Eligibility Criteria

The review will include Studies that have developed or evaluated interventions to target the communication of musculoskeletal imaging findings. Studies in any healthcare setting worldwide and published in any language will be included, where languages other than English will be translated. This review will include not limit studies by publication year. Studies that include multiple interventions or broad treatment approaches will be included if it was possible to isolate specific BCTs intended to affect the communication of imaging findings within them. Studies using qualitative methods to develop interventions will be included if their findings identify a target for interventions. The review will exclude studies focussing on serious or specific known causes of musculoskeletal pain such as fracture, malignancy, infection and inflammatory arthritis.

This scoping review will consider both experimental and quasi-experimental study designs including randomized controlled trials, non-randomized controlled trials, before and after studies and interrupted time-series studies, cluster randomised trials, non-randomised cluster trials, controlled and uncontrolled before-after studies and cross sectional studies.

Information sources and Search strategy

For peer review only - http://bmjopen.bmj.com/site/about/guidelines.xhtml

The search strategy will aim to locate published studies. A three-step search strategy will be utilized in this review. First, an initial limited search of MEDLINE (PubMed) and CINAHL (EBSCO) was undertaken to identify articles on the topic. The text words contained in the titles and abstracts of relevant articles, and the index terms used to describe the articles will be used to develop a full search strategy for MEDLINE, EMBASE, CINAHL, AMED and PsycINFO (see Appendix I). The search will be performed in July 2023. We will email experts in the field of imaging and LBP to identify any studies that may have been missed by the search. The search strategy, including all identified keywords and index terms, will be adapted for each database and/or information source. The reference list of all included sources of evidence will be screened for additional studies. A specialist healthcare librarian will review the search strategy.

Study selection

Following the search, all identified citations will be collated and uploaded into Refworks [Proquest LLC, Michigan USA] and duplicates removed. Following a pilot test, titles and abstracts will then be screened by two or more independent reviewers for assessment against the inclusion criteria for the review. Potentially relevant sources will be retrieved in full and assessed in detail against the inclusion criteria by two or more independent reviewers. Reasons for exclusion of papers at full text that do not meet the inclusion criteria will be recorded and reported in the systematic review. Any disagreements that arise between the reviewers at each stage of the selection process will be resolved through discussion, or with an additional reviewer/s. The results of the search and the study inclusion process will be reported in full in the final systematic review and presented in a Preferred Reporting Items for Systematic Reviews and Meta-analyses (PRISMA) flow diagram (26)

Assessment of Methodological Quality

as the objective of the study is to determine which Behaviour Change Techniques (BCT's) have been employed, what the behavioural targets were and which behavioural theories and theoretical models underpinned the interventions designed to affect MSK image reporting, the outcomes and therefore assessment of methodological quality is not considered necessary and will not be performed.

Data extraction

Two reviewers will independently extract the study characteristics (year, country, setting, design, patient numbers (n), MSK area, outcome measures, effect sizes barriers, enablers, confounders and modifiers) and intervention information using the Template for Intervention Description and Replication (TIDieR). (27)

The TIDieR template will be used to characterise the intervention. A draft coding manual will be created based on the BCT taxonomy v1 [see Appendix II] and piloted. Coding will based on principles outlined in Lorencato et al (2013). (28) The draft data extraction tool will be modified and revised as necessary during the process of extracting data from each included evidence source. Any coding

For peer review only - http://bmjopen.bmj.com/site/about/guidelines.xhtml

disagreements that arise between the reviewers will be resolved through discussion, or with a third reviewer. Authors of papers will be contacted to request missing or additional data, where required.

The Theory and Techniques Tool (TATT) (available at: https://theoryandtechniquetool. humanbehaviourchange.org/tool) will be used to map BCTs to their corresponding mechanisms of action in the TDF. The Theory and Techniques tool is a map of 74 BCTs linking to 26 mechanisms of action and attributing strength to the association based on research and expert consensus.

Theories explicitly mentioned that either inform the intervention or where the intervention tests or creates theory will be extracted. Item 2 on the Tidier template – "Why: describe the use of any rationale, theory or goal of the elements essential to the intervention" will be used to extract both evidence of behavioural theory used and context specific models. The Painter criteria (29) will then be applied to the details extracted. This categorisation [see Appendix III] distinguishes the use of theory into the following categories: (i) informed by theory, (ii) applied theory, (iii) testing theory or (iv) building or creating theory.

Data presentation

The Study characteristics, BCT's identified, behavioural targets (according to the COM-B) and context specific theories within the studies will be presented in narrative and table form. An example table which will be used to extract the BCT's is included in Appendix IV and a table which will be used to map the behavioural targets to the COM-B components is included in Appendix V.

Ethics and dissemination

This review will be the first step to formally identify which BCT's have been employed, what the behavioural targets were and which behavioural theories and theoretical models underpinned the interventions designed to affect MSK image reporting. This information will be used to inform an intervention design process seeking to improve the communication of imaging results. The results will be disseminated through a peer-reviewed publication, conference presentations and stakeholder events.

Patient and Public Involvement (PPI)

Patients with experience of receiving MSK imaging reports were invited to participate in individual sessions initially by advertising in person and using study flyers within radiology departments and GP surgeries. These initial sessions sought to explore peoples' experience of imaging report communication and the ways that this could be improved, such as the setting, personnel involved and resources that would be helpful to them. These meetings highlighted clinical behaviours that were discordant with patient preference. Based on this information it was deemed necessary to investigate the barriers to providing helpful communication of reports and the current review of whether existing interventions had targeted these. Further PPI group sessions are planned to discuss the results of this review and to have input into the design and implementation of further work seeking to improve the communication of MSK imaging findings.

For peer review only - http://bmjopen.bmj.com/site/about/guidelines.xhtml

Author Contributions

EK designed and wrote the protocol with planning and design input from BL and ABr and ABa. Studies will be screened by EK and AM. Data will be extracted by EK and AM. Data will be coded by EK and AB. AM, BL, ABr and Aba will review and make amendments to drafts of the manuscript and approve the final draft.

Funding

EK is an ICA Pre-doctoral Clinical and Practitioner Academic Fellow supported by Health Education England and the National Institute for Health and Care Research. The University of Essex have provided funding for associated Article Processing Charges.

For peer review only

Declarations

The views expressed in this publication are those of the author(s) and not necessarily those of the NHS, the National Institute for Health and Care Research or the Department of Health and Social Care

Conflicts of interest

There is no conflict of interest in this project

References

1. Wu A, March L, Zheng X, Huang J, Wang X, Zhao J, et al. Global low back pain prevalence and years lived with disability from 1990 to 2017: estimates from the Global Burden of Disease Study 2017. Annals of translational medicine. 2020;8(6).

2. Global Burden of Disease Collaborative Network. Global Burden of Disease Study 2017 (GBD 2017) Results. Seattle: Institute for Health Metrics and Evaluation (IHME); 2018.

3. National Institute for Health and Care Excellence. Osteoarthritis in over 16s: diagnosis and management [Internet]. UK: National Institute for Health and Care Excellence; 2022 [cited 2022 Nov 17]. Available from: https://www.nice.org.uk/guidance/ng226

4. National Institute for Health and Care Excellence 2016. Low back pain and sciatica in over 16s: assessment and management [Internet]. UK: National Institute for Health and Care Excellence.; 2016 [cited 2022 Nov 17]. Available from: https://www.nice.org.uk/guidance/ng59

5. Lin IB, O'Sullivan PB, Coffin JA, Mak DB, Toussaint S, Straker LM. Disabling chronic low back pain as an iatrogenic disorder: a qualitative study in Aboriginal Australians. BMJ open. 2013;3(4).

For peer review only - http://bmjopen.bmj.com/site/about/guidelines.xhtml

6. Slade SC, Molloy E, Keating JL. The dilemma of diagnostic uncertainty when treating people with chronic low back pain: a qualitative study. Clinical rehabilitation. 2012;26(6):69.

7. Sharma S, Traeger AC, Reed B, Hamilton M, O'Connor DA, Hoffmann TC, et al. Clinician and patient beliefs about diagnostic imaging for low back pain: a systematic qualitative evidence synthesis. BMJ open. 2020;10(8).

8. Farre A, Rapley T. The New Old (and Old New) Medical Model: Four Decades Navigating the Biomedical and Psychosocial Understandings of Health and Illness. Healthcare (Basel, Switzerland). 2017;5(4).

9. Brinjikji W, Luetmer PH, Comstock B, Bresnahan BW, Chen LE, Deyo RA, et al. Systematic literature review of imaging features of spinal degeneration in asymptomatic populations. AJNR American journal of neuroradiology. 2015;36(4):6.

10. Horga LM, Hirschmann AC, Henckel J, Fotiadou A, Di Laura A, Torlasco C, et al. Prevalence of abnormal findings in 230 knees of asymptomatic adults using 3.0 T MRI. Skeletal radiology. 2020;49(7):1107.

11. Barreto RPG, Braman JP, Ludewig PM, Ribeiro LP, Camargo PR. Bilateral magnetic resonance imaging findings in individuals with unilateral shoulder pain. Journal of shoulder and elbow surgery. 2019;28(9):1706.

12. O'Keeffe M, Ferreira GE, Harris IA, Darlow B, Buchbinder R, Traeger AC, et al. Effect of diagnostic labelling on management intentions for non-specific low back pain: A randomized scenario-based experiment. European journal of pain (London, England). 2022;26(7):1545.

13. Malliaras P, Rathi S, Burstein F, Watt L, Ridgway J, King C, et al. "Physio's not going to repair a torn tendon": patient decision-making related to surgery for rotator cuff related shoulder pain. Disability and rehabilitation. 2022;44(14):3693.

14. Bunzli S, Watkins R, Smith A, Schutze R, O'Sullivan P. Lives on hold: a qualitative synthesis exploring the experience of chronic low-back pain. The Clinical journal of pain. 2013;29(10):16.

15. Ash LM, Modic MT, Obuchowski NA, Ross JS, Brant-Zawadzki MN, Grooff PN. Effects of diagnostic information, per se, on patient outcomes in acute radiculopathy and low back pain. AJNR American journal of neuroradiology. 2008;29(6):103.

16. Graves JM, Fulton-Kehoe D, Jarvik JG, Franklin GM. Health care utilization and costs associated with adherence to clinical practice guidelines for early magnetic resonance imaging among workers with acute occupational low back pain. Health services research. 2014;49(2):65.

17. Rajasekaran S, Dilip Chand Raja S, Pushpa BT, Ananda KB, Ajoy Prasad S, Rishi MK. The catastrophization effects of an MRI report on the patient and surgeon and the benefits of "clinical reporting": results from an RCT and blinded trials. European spine journal : official

For peer review only

publication of the European Spine Society, the European Spinal Deformity Society, and the European Section of the Cervical Spine Research Society. 2021;30(7):2081.

18. Jarvik JG, Meier EN, James KT, Gold LS, Tan KW, Kessler LG, et al. The Effect of Including Benchmark Prevalence Data of Common Imaging Findings in Spine Image Reports on Health Care Utilization Among Adults Undergoing Spine Imaging: A Stepped-Wedge Randomized Clinical Trial. JAMA network open. 2020;3(9).

19. Witherow JL, Jenkins HJ, Elliott JM, Ip GH, Maher CG, Magnussen JS, et al. Characteristics and Effectiveness of Interventions That Target the Reporting, Communication, or Clinical Interpretation of Lumbar Imaging Findings: A Systematic Review. AJNR American journal of neuroradiology. 2022;43(3):500.

20. Skivington K, Matthews L, Simpson SA, Craig P, Baird J, Blazeby JM, et al. A new framework for developing and evaluating complex interventions: update of Medical Research Council guidance. BMJ (Clinical research ed). 2021;374.

21. Michie S AL. The Behaviour Change Wheel - A Guide to Designing Interventions. 1st ed. Great Britain: Silverback Publishing; 2014. 329 p.

For peer review only

For peer review only - http://bmjopen.bmj.com/site/about/guidelines.xhtml

22. Hall A, Richmond H, Pike A, Lawrence R, Etchegary H, Swab M, et al. What behaviour change techniques have been used to improve adherence to evidence-based low back pain imaging?. Implementation science : IS. 2021;16(1).

23. Michie S, Hyder N, Walia A, West R. Development of a taxonomy of behaviour change techniques used in individual behavioural support for smoking cessation. Addictive behaviors. 2011;36(4):9.

24. Aromataris E MZ. J. JBI Manual for Evidence Synthesis. 1st ed. Australia: JBI; 2020.

25. Tricco AC, Lillie E, Zarin W, O'Brien KK, Colquhoun H, Levac D, et al. PRISMA Extension for Scoping Reviews (PRISMA-ScR): Checklist and Explanation. Annals of internal medicine. 2018;169(7):473.

26. Moher D, Liberati A, Tetzlaff J, Altman DG, PRISMA Group. Preferred reporting items for systematic reviews and meta-analyses: the PRISMA statement. BMJ (Clinical research ed). 2009;339.

27. Hoffmann TC, Glasziou PP, Boutron I, Milne R, Perera R, Moher D, et al. Better reporting of interventions: template for intervention description and replication (TIDieR) checklist and guide. BMJ (Clinical research ed). 2014;348.

28. Lorencatto F, West R, Seymour N, Michie S. Developing a method for specifying the components of behavior change interventions in practice: the example of smoking cessation. Journal of consulting and clinical psychology. 2013;81(3):44.

29. Painter JE, Borba CPC, Hynes M, Mays D, Glanz K. The use of theory in health behavior research from 2000 to 2005: A systematic review. Annals of Behavioral Medicine [Internet]. 2008 Jun;35(3):362. Available from: https://www.proquest.com/scholarly-journals/use-theory-health-behavior-research-2000-2005/docview/837460954/se-2?accountid=47999

For peer review only

Appendices

Appendix I: Search strategy

Ovid MEDLINE(R) ALL <1946 to December 14, 2022>

1	exp Musculoskeletal Pain/ or musculoskeletal.mp.
2	exp Patellofemoral Pain Syndrome/ or exp Back Pain/ or exp Low Back Pain/ or exp Shoulder Pain/ or exp Neck Pain/
3	osteoarthriti*.mp. or exp Osteoarthritis, Hip/ or exp Osteoarthritis/ or exp Osteoarthritis, Spine/ or exp Osteoarthritis, Knee/
4	spondylosis.mp. or exp Spondylosis/
5	(backache or lumbago or radicular or sciatica).mp.
6	("leg pain" or "foot pain" or "heel pain" or "ankle pain" or "knee pain" or "patellofemoral pain" or "hip pain" or "arm pain" or "shoulder pain" or "subacromial pain" or "elbow pain" or "hand pain" or "wrist pain").mp.
7	exp chronic pain/ or "chronic pain".mp. or "persistent pain".mp.
8	exp Humans/
9	exp Animals/
10	8 and 9
11	9 not 10
12	"diagnostic imaging".mp. or exp Diagnostic Imaging/
13	exp Radiography/ or radiograph*.mp.
14	IMAGING.mp. or exp Magnetic Resonance Imaging/ or "magnetic resonance".mp. or MRI.mp.
15	exp Radiology/ or exp Radiologists/ or RADIOLOG*.mp. or exp Radiology Information Systems/
16	Tomography, X-Ray Computed/ or "COMPUTED TOMOGRAPHY".mp.
17	CT.mp.
18	(ultrasound or ultrasonograph*).mp.

For peer review only

19	exp X-Rays/
20	X?RAY.mp.
21	exp Education, Medical/ or exp Interprofessional Education/ or exp Patient Education Handout/ or exp Patient Education as Topic/ or exp Health Education/
22	exp Health Communication/
23	"HEALTH COMMUNICATION".mp.
24	"REPORTING STRATEG*".mp.
25	(EPIDEMIOLOGIC* adj2 (DATA or INFORMATION)).mp.
26	(PREVALENCE adj2 INFORMATION).mp
27	(PAIN adj2 EDUCATION).mp.
28	("pain neurobiology" or "pain neurophysiology").mp.
29	Patient Education as Topic/ or PSYCHOEDUCATION*.mp.
30	(COMMUNICAT* adj5 ("IMAGing FINDINGs" or "IMAGing RESULTs" or "imaging report" or " MRI findings" or "MRI results" or "MRI REPORT*" or "X-RAY findings" or "X-Ray results" or "X-Ray REPORT" or "CT findings" or "CT REPORT" or "CT results" or "RADIOLOG* FINDINGS*" or "radiolog* results" or "radiolog* report")).
31	((WRITTEN or PRINTED or ORAL) adj INFORMATION).mp.
32	(interpretation adj3 ("IMAGing FINDINGs" or "IMAGing RESULTs" or "imaging report" or " MRI findings" or "MRI results" or "MRI REPORT*" or "X-RAY findings" or "X-Ray results" or "X-Ray REPORT" or "CT findings" or "CT REPORT" or "CT results" or "RADIOLOG* FINDINGS*" or "radiolog* results" or "radiolog* report")).mp.
33	(REASSUR* adj3 ("IMAGing FINDINGs" or "IMAGing RESULTs" or "imaging report" or " MRI findings" or "MRI results" or "MRI REPORT*" or "X-RAY findings" or "X-Ray results" or "X-Ray REPORT" or "CT findings" or "CT REPORT" or "CT results" or "RADIOLOG* FINDINGS*" or "radiolog* results" or "radiolog* report")).mp.
34	(CONTEXTUALI* adj3 ("IMAGing FINDINGs" or "IMAGing RESULTs" or "imaging report" or " MRI findings" or "MRI results" or "MRI REPORT*" or "X-RAY findings" or "X-Ray results" or "X-Ray REPORT" or "CT findings" or "CT REPORT" or "CT results" or "RADIOLOG* FINDINGS*" or "radiolog* results" or "radiolog* report")).mp
35	((perception or perceive) adj3 ("IMAGing FINDINGs" or "IMAGing RESULTs" or "imaging report" or " MRI findings" or "MRI results" or "MRI REPORT*" or "X-RAY findings" or "X-Ray

For peer review only

results" or "X-Ray REPORT" or "CT findings" or "CT REPORT" or "CT results" or "RADIOLOG* FINDINGS*" or "radiolog* results" or "radiolog* report")).mp.

36 (DIAGNOS* adj2 INFORMATION).mp.

37 (INTERVENTION* adj5 ("IMAGing FINDINGs" or "IMAGing RESULTs" or "imaging report" or " MRI findings" or "MRI results" or "MRI REPORT*" or "X-RAY findings" or "X-Ray results" or "X-Ray REPORT" or "CT findings" or "CT REPORT" or "CT results" or "RADIOLOG* FINDINGS*" or "radiolog* results" or "radiolog* report")).mp

38 BIOPSYCHOSOCIAL.mp.

39 (behavio?r adj2 intervention).mp.

40 "behavio?r change".mp.

41 exp cognitive behavioral therapy/ or "cognitive behavio?ral therapy".mp.

42 "cognitive functional therapy".mp.

43 exp Arthritis, Rheumatoid/

44 exp Neoplasms/ or sarcoma*.mp. or osteosarcoma*.mp. or tumo?r*.mp.

45 exp Musculoskeletal Diseases/cn, su [Congenital, Surgery]

46 exp Central Nervous System/

47 exp Central Nervous System Diseases/

48 exp Dentistry/

49 exp Tooth Diseases/

50 1 or 2 or 3 or 4 or 5 or 6 or 7

51 12 or 13 or 14 or 15 or 16 or 17 or 18 or 19 or 20

52 21 or 22 or 23 or 24 or 25 or 26 or 27 or 28 or 29 or 30 or 31 or 32 or 33 or 34 or 35 or 36 or 37 or 38 or 39 or 40 or 41 or 42

53 43 or 44 or 45 or 46 or 47 or 48 or 49

54 50 and 51 and 52

55 54 not 53

56 55 not 11

For peer review only

Appendix II: Data Extraction - Coding Rules

1. Patient as mechanism of action
   If the patient is given something that relates to the behaviour in question, e.g. a letter aiming to promote the robustness of the spine and benefits of movement, that they need to take with them to their appointment to discuss with their HCP code as: 7.1 - "Prompts/cues" Or 12.1 - "restructuring social environment" dependent on the format and extent of the information.

2. Patient education materials
   If patient education materials are delivered to an HCP to use with patients, then code as

   12.5 "adding objects to environment" and add additional code explaining nature of information eg. 5.1 information about health consequences, 7.1 "prompt/cue (poster)", 9.2 "pro and cons"

   Consider additional coding if information comes with support eg. 3.2 - "social support"

   If the research team indicates that the patient education material eg. Booklet was intended to act as a reminder then also code as 7.1 - "Prompt cue"

   If Patient education material delivered directly to patients from researchers (or someone other than the clinician) – do not code.

3. HCP Training
   Code as 4.1 – "instruction on how to perform a behaviour"

   If involves practice then additionally code as 8.1 – "behavioural practice/rehearsal"

   Only code as 6.1 demonstration of the behaviour and 8.1 behavioural practice/rehearsal if the intervention explicitly states demonstration.

4. Information provided to clinicians (relates closely to education and persuasion)
   Prospective information e.g. "The consequence of harmful reporting is increasing fear and downstream costs" Code as 5.1 – "information about health consequences".

   If information about emotional consequences code as 5.6 " information about emotional cosequences"

   For retrospective information code as: 2.7 – "feedback on outcomes of behaviour"

5. Amending/Withholding imaging findings (relates closely to restructuring and enablement)
   12.1 Restructuring the physical environment

   May also involve 12.3 avoidance/reducing cues for the behaviour

   12.5 if adding objects to the environment

6. Inclusion of case studies/patient vignettes
   Code as 4.1 - "instruction on how to perform a behaviour unless there was explicit evidence of OR 6.1 "Demonstration of the behaviour" or 8.1 – "Behavioural Practice/Rehearsal"

For peer review only

Appendix III: Categorisation the use of theory (Painter et al, 2008)

1 - Informed by theory:

Theoretical framework or construct identified but no or limited/partial application of theoretical framework in study components and measures. For example, one study stated that the Health Belief Model (HBM) was used to develop intervention materials, but no applications of HBM constructs were explicitly described, and no HBM constructs were measured.

2 - Applied theory:

Theoretical framework specified and between one and half of the constructs applied in components of the study. An example was a study that described intervention materials as based on the HBM and addressed cues to action and perceived threat.

3 - Testing theory:

Theoretical framework specified; more than half of the theoretical constructs in intervention or descriptive/explanatory research were measured and explicitly tested, or two or more theories were explicitly compared to one another in the study. For example, a study stated that the HBM was used to develop intervention materials. The study used and measured five key constructs in the intervention and evaluation: perceived benefits and barriers, perceived susceptibility and severity, and cues to action. Mediation analyses were conducted with indicators of these constructs.

4 - Building or creating theory:

Developing new or revised/expanded theory using constructs specified, measured, and analysed in a study. For example, a study stated that a new hybrid theory was created to develop intervention materials. The new theory integrated HBM constructs, such as perceived benefits and barriers with other constructs such as group involvement and social norms. The study tested the intervention effects and the hypothesized theoretical constructs' mediating effects and examined each construct's contribution to the new extended theoretical framework.

It is worth noting that our determination of theory use along the continuum [will rely] on analysis of the article as published rather than the article's stated intent, title, or abstract. For example, if the authors of a given article claimed that they were applying a theory but only measured one construct, it would have been included in the "informed" by theory category. Stages of change are one core construct of the Transtheoretical Model (TTM), so if an article only used the stages of change construct from TTM, then it was "informed" by TTM [31].

For peer review only

Appendix IV. Example template for illustrating extracted BCT's

Author	Target: Patient/clinician	Action Planning	Instruction on how to perform behaviour	Information about health consequences	Demonstration of the behaviour	Prompts/cues	Behavioural practice/Rehearsal	Credible source	Avoidance/reducing exposure to cues for the behaviour	Adding objects to the environment	Framing/Reframing	Incompatible beliefs	Number of BCT's

Appendix V. Example template for illustrating relationship between BCT's and COM-B components

BCT	Author	Capability				Opportunity		Motivation					
		Physical	Psychological			Social	Physical	Reflective					Automatic
		Skills	Knowledge	Memory attention and decision processes	Behavioural regulation	Social influences	Environmental content and resources	Beliefs about capabilities	Beliefs about consequences	Social professional role and identity	optimism	goals	Emotions

For peer review only
